# Advances in Magnetic Noble Metal/Iron-Based Oxide Hybrid Nanoparticles as Biomedical Devices

**DOI:** 10.3390/bioengineering6030075

**Published:** 2019-08-28

**Authors:** Laura M. Sanchez, Vera A. Alvarez

**Affiliations:** Materiales Compuestos Termoplásticos (CoMP), Instituto de Investigaciones en Ciencia y Tecnología de Materiales (INTEMA), CONICET-Universidad Nacional de Mar del Plata (UNMdP). Av. Colón 10850, Mar del Plata 7600, Argentina

**Keywords:** nanoparticles, hybrid materials, magnetic, noble metal, biomedicine

## Abstract

The study of the noble metal magnetic hybrid nanoparticles is a really promising topic from both the scientific and the technological points of views, with applications in several fields. Iron oxide materials which are hybridized with noble metal nanoparticles (NPs) have attracted increasing interest among researchers because of their cooperative effects on combined magnetic, electronic, photonic, and catalytic activities. This review article contains a summary of magnetic noble metal/iron oxide nanoparticle systems potentially useful in practical biomedical applications. Among the applications, engineered devices for both medical diagnosis and treatments were considered. The preparation to produce different structures, as blends or core-shell structures, of several nanometric systems was also considered. Several characterization techniques available to describe the structure, morphology and different kinds of properties of hybrid nanoparticles are also included in this review.

## 1. Introduction 

Recently, huge progress in the field of nanobiotechnology towards the development of different kinds of nanomaterials with a wide range of applications has been made [1,2]. The study of nanoscale particles has been generally related to changes in chemical and physical properties as compared to bulk materials [3]. In this context, nanomaterials have attracted growing interest in several applications and particularly in biomedicine due to their potentiality to revolutionize the diagnosis and treatment of many diseases [4]. Owing to the small size, nanoparticles (NPs) can easily interact with biomolecules thus generating a nano-bio interface comprising the kinetics, dynamic interactions and thermodynamic exchanges between nanomaterial surfaces and the corresponding surfaces of the biological components (such as proteins, phospholipids and so on) [5]. In this sense, it is important to remark that the size and other characteristics/properties of the NPs can be conveniently engineered, making them relevant for application in both in vivo and in vitro biomedical fields [6,7,8]. 

It is known that noble metals, and compounds based on them, have been used as therapeutic agents from the ancient time in medicine for the treatment of different infections. Noble metal NPs have inspired the researchers due to their remarkable role in detection and treatment of dreadful diseases [9]. 

Some important characteristics of noble metals are related to their exceptional resistance to corrosion (for a wide range of liquid and gaseous substances) and their stability at high temperatures under conditions where base metals are rapidly oxidized [9]. Noble metals could be considered as unreactive elements; they does not form compounds with other substances; thus having multiple applications [9]. Noble metals mainly include silver, gold, platinum, palladium, rhodium, rhenium, ruthenium, iridium, and osmium. Many efforts have been made in order to produce and characterize nanoparticles from noble metal and also to investigate their processing–size–shape–properties–behaviour relationships [10,11,12]. 

Among all metal materials, noble and magnetic metals are two groups of the most promising materials. Magnetic iron oxides (mainly magnetite and maghemite) are principally attractive as components of multifunctional nanomaterials because they interact with external fields, giving them the ability to be easily recovered and reused or otherwise manipulated by magnetic means [13]. Superparamagnetic iron oxide NPs have multiple functionalities that make them very attractive for biomedical applications [14]. Magnetic manipulation has been successfully proposed for drug targeting in specific locations of the body, to enhance cell therapy by retaining therapeutic cells on the site to be regenerated or for tissue engineering in order to reproduce the native cellular organization of tissues. In contrast, severe restrictions are related to the use of magnetic nanoparticles (MNPs) for in vivo biomedical applications [14]. Some of them are related to the organs involved in NPs metabolism (lungs, liver, spleen, and kidney), others with the chemical and physical characteristics of MNPs that can influence their pharmacokinetics (absorption, biodistribution and elimination), targeting ability, and optical properties [15]. It is known that nanometric iron oxides can be synthesized by several routes, such as thermal decomposition, co-precipitation and solvothermal methods [16]. 

Multifunctional NPs are more interesting than their single-component analogues since they have the potential to combine the unique properties of two or more nanomaterials in one entity. This concept has led to advances in biomedical technologies, for example in the development of dual-action theranostics [13]. In particular, by combining iron oxides and noble metals to generate new useful nanomaterials allows having devices that offer properties enabling them to conduct more than two simultaneous treatments, like magnetic hyperthermia and photothermia [17], thus easily improving the efficiency of the medical treatment. 

**Figure 1:**
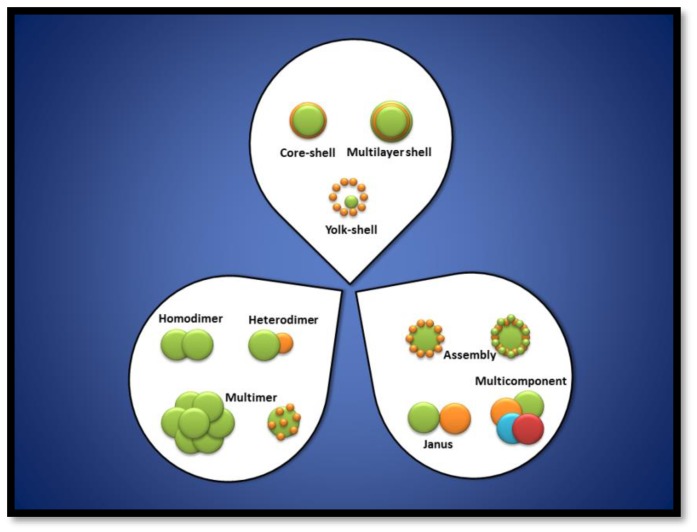
Different possibilities for metal oxides (MO)/noble metal nanohybrid materials.

The overall architecture of nanocomposites is one of the most important factors dictating the physical properties of nanohybrids. Noble metals can be coupled to metal oxides to yield diversified nanostructures, including noble metal decorated-metal oxide NPs, nanoarrays, noble metal/metal oxide core/shell, noble metal/metal oxide yolk/shell and Janus noble metal–metal oxide nanostructures [18]. Some relevant configurations of metal oxides (MO)/noble metals nanohybrids are included in Figure 1. Nevertheless, the control on hybrid materials characteristics and properties is difficult and it usually involves methodologies of known multistep organic synthesis. It is required that the obtaining conditions for one material do not harm or interfere with the properties of the second material [19]; so that classic protocols to prepare these kinds of hybrid systems can be not only time-consuming and tedious but also specific to a single system and not extrapolable to other one [19].

The aim of this review was to summarize magnetic noble metal/iron oxide nanoparticle systems potentially useful in practical biomedical applications. The synthesis, characterization and applications of such materials are included in this review. Engineered devices for both medical diagnosis and treatments were considered. The preparation to produce several kinds of nanometric systems was also approached, taking into account the importance of studying their processing–size–shape–properties–behaviour relationships [10,11,12]. 

## 2. Magnetic Noble Metal Nanoparticles (NPs)

### 2.1. Methods for the Synthesis of Magnetic Noble Metal Nanoparticles 

**Figure 2:**
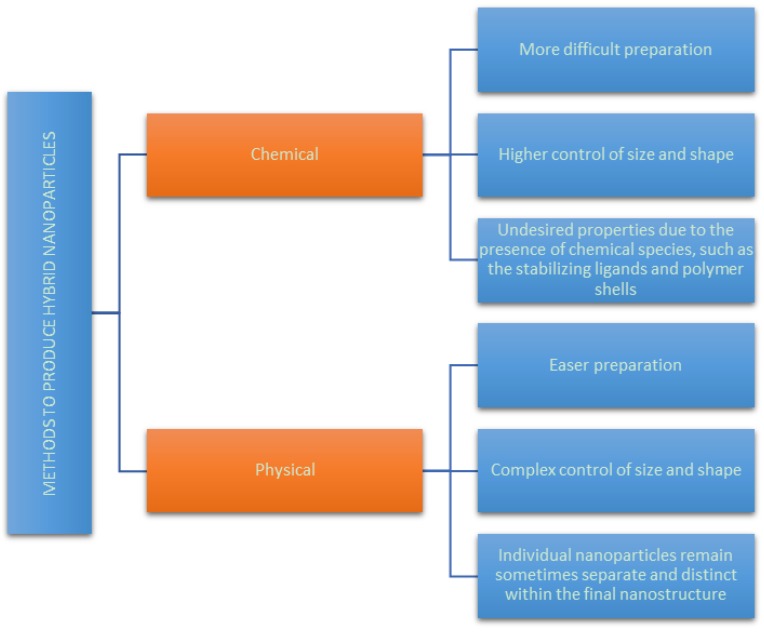
Advantages and disadvantages of the chemical and physical methods available to produce hybrid nanoparticles.

Generally, synthetic methods can be classified into two main categories: chemical and physical. Figure 2 resumes the advantages and disadvantages of the available chemical and physical methods to produce hybrid nanoparticles. 

**Figure 3:**
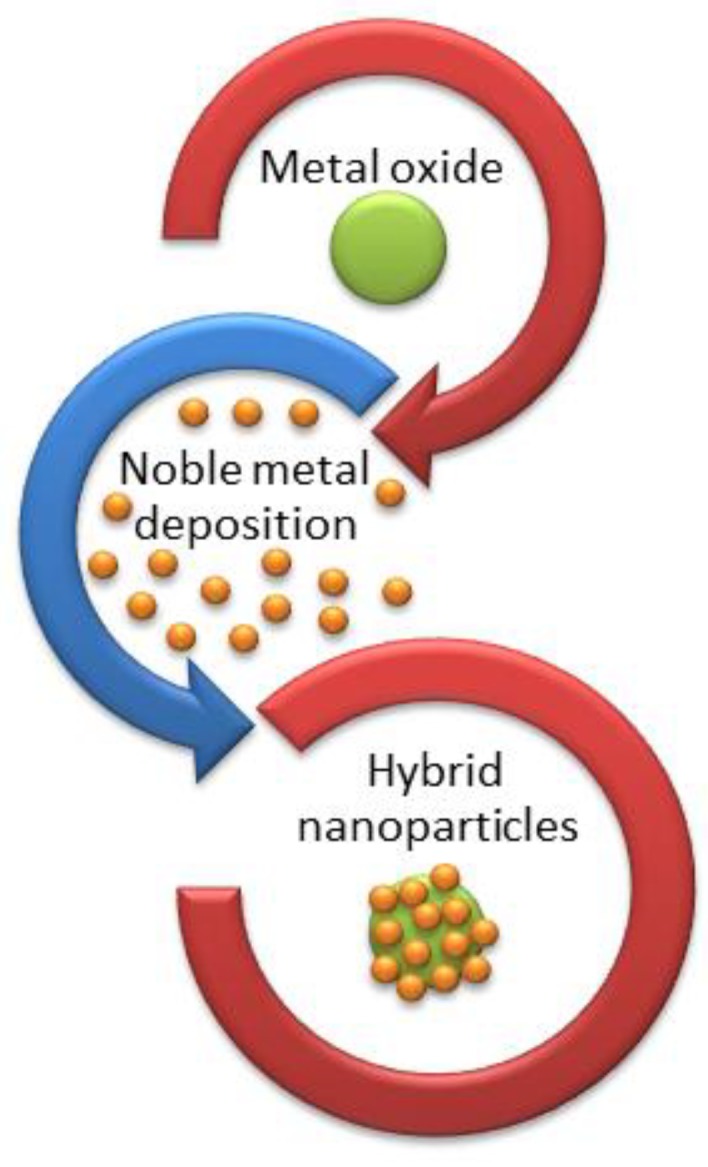
Schematic representation of chemical reduction (CR) method for the production of OM/noble metal hybrid nanoparticles.

Among the chemical methods, one possibility is to separate them in two main categories: the reduction of a metal precursor as chemical reduction (CR) and photoreduction methods. These methods are commonly used when noble metal NPs are deposited on the surface of MO NPs, resulting in noble metal-decorated MO NPs. In the chemical reduction method, some reducing agents [20] are required for the synthesis of noble metal/MO hybrid NPs [21] , as shown in Figure 3. 

The disadvantages of this kind of method are mainly related to the obtaining of a mixture of both pure noble metal nanoparticles and hybrid nanoparticles. Nevertheless, three different strategies are available in order to solve this drawback. They are based on the use of: a) local immobilization of chemical reducing agents on the surface of MO nanoparticles [22]; b) a redox reaction of metal hydroxide and noble metal ion (without reducer) [23] and c) a low weight ratio of noble metals precursors and MO (around 1:30). In photoreduction methods, photoelectrons from MO play an important role during the reducing process upon light irradiation.

**Figure 4:**
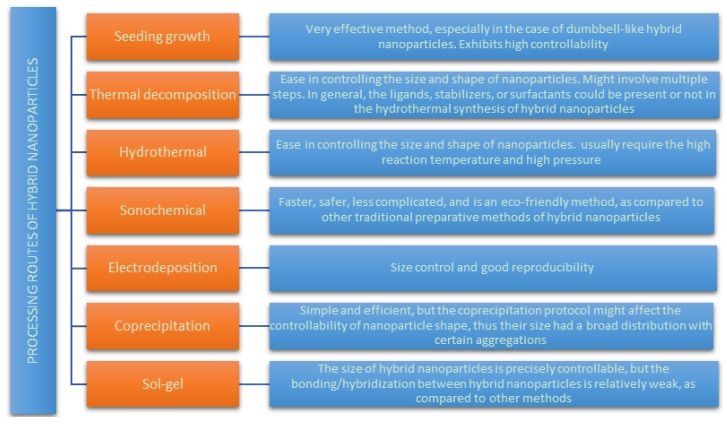
Processing routes, with their main characteristics, used to fabricate hybrid nanoparticles by chemical methods.

On the other hand, several processing routes [24] can be used to fabricate hybrid nanoparticle. The most common processing routes used to fabricate hybrid nanoparticles by chemical methods are summarized in Figure 4. The main characteristic of each method are also included in this Figure. 

The previous figure clearly shows that each technique used to produce hybrid nanoparticles has differential characteristics and displays some advantages and disadvantages. The selection of a specific technique will be based on several factors such as the kind of desired structure, the necessity of size control, the times available to produce the particles, the required interaction between both components (noble metal and MO), among others. 

**Figure 5:**
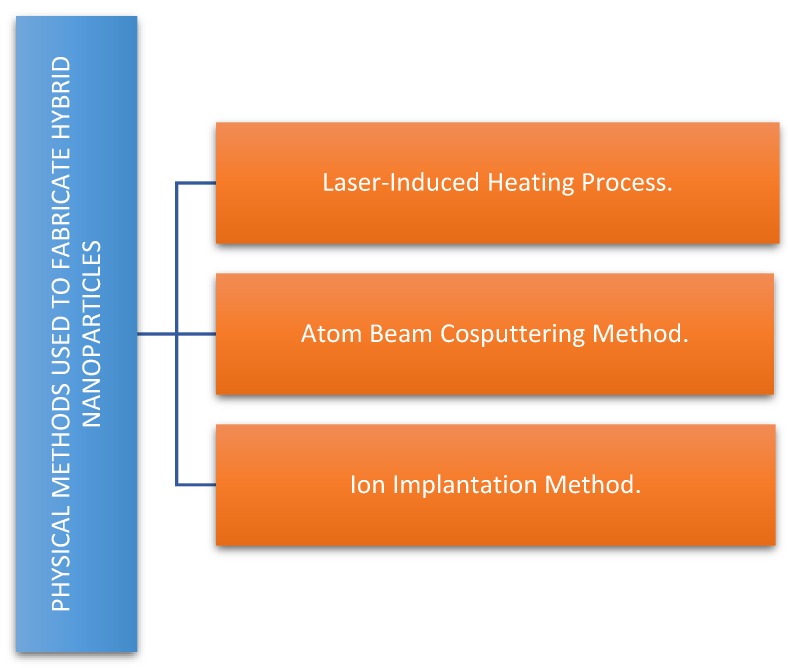
Main physical methods used to fabricate hybrid nanoparticles.

The most relevant physical methods used to produce hybrid nanoparticles are summarized in Figure 5.

**Figure 6:**
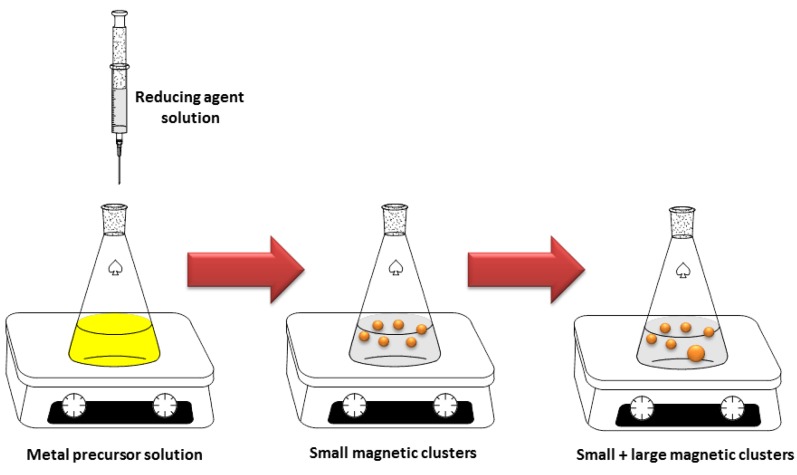
Schematic representation of an alternative and easy method to produce OM/noble metal NPs.

Additionally, a different option can be taken in order to produce MO/noble metal NPs. When conventional method of making these NPs is carried out under a strong magnetic field, it turns out that the nanoparticles, or clusters of nanoparticles, actually become magnetic themselves (Figure 6). The apparatus and reagents needed for making magnetic noble metal are so trivial that any decently equipped scientific laboratory should be able to reproduce the reported results [25].

The aforementioned process, developed at the TU Delft, is different from previous approaches in the aspects that the size of particles is not limited (magnetic nano and millimetric sized particles can be made) and also that capping agents are not necessary. By excluding the possibility that the identified magnetism is actually caused by impurities in the particles, the researchers have proven that intrinsic magnetism can be invoked in noble metals, some of which are even close to satisfying Stoner's criterion for ferromagnetism [25].

### 2.2. Nanoscale Characterization of Magnetic Noble Metal Nanoparticles

**Figure 7:**
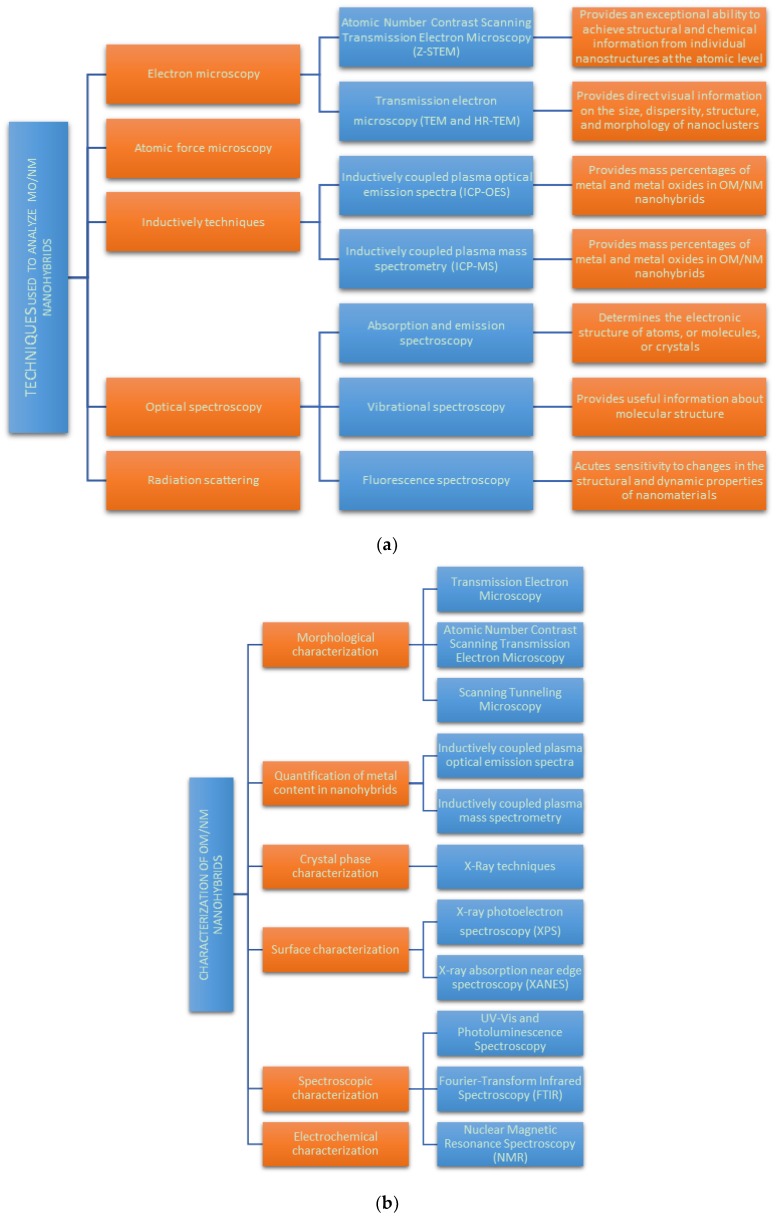
(**a**) Relevant techniques used to characterize MO/noble metal nanohybrids (classified as families of techniques). (**b**) Characterization techniques of MO/noble metal hybrid nanoparticles (classified by characterization type).

Once MO/noble metal hybrid nanoparticles are produced it is important to characterize them in order to understand their structure and properties. It is important to mention that most of the methods for the characterization of hybrid nanomaterials are already in development [26]. General characterization techniques are useful to understand the morphology for controlled formation of MO/noble metal nanohybrids. Some of the most relevant techniques used to characterize them are included in Figure 7a,b.

The electron and atomic force microscopy, optical spectroscopy and radiation scattering techniques are widely used. Applying these techniques to measure nanoparticles sizes, their structure, and also their composition can help to understand the underlying synthetic mechanism. The high resolution transmission electron microscopy (HRTEM) coupled with energy-dispersive X-ray spectroscopy (EDS) constitutes an essential tool for the structural characterization of the MO/noble metal nanohybrids providing their lattice parameters and revealing their crystal structure [27]. 

The surface characterization is mainly due by X-ray photoelectron spectroscopy (XPS) and X-ray absorption near edge spectroscopy (XANES). X-ray photoelectron spectroscopy (XPS) is a surface-sensitive quantitative spectroscopic technique that measures the elemental composition at the parts per thousand range, empirical formula, chemical state and electronic state of the elements that exist within a material. (XANES), also known as near edge X-ray absorption fine structure (NEXAFS), is a type of absorption spectroscopy that indicates the features in the X-ray absorption spectra (XAS) of condensed matter due to the photoabsorption cross section for electronic transitions from an atomic core level to final states in the energy region of 50–100 eV above the selected atomic core level ionization energy, where the wavelength of the photoelectron is larger than the interatomic distance between the absorbing atom and its first neighbour atoms [28]. It is also important to mention that EDS can provide elemental analysis, with both composition and mapping capabilities available on many instruments.

Spectroscopic characterization includes ultraviolet–visible (UV–Vis) and photoluminescence spectroscopy and Fourier transform infrared spectroscopy (FTIR). UV–Vis spectroscopy is absorption spectroscopy in the UV and visible portion of the electromagnetic spectrum. Molecules having non-bonding electrons can absorb the energy in the form of UV or visible light to excite these electrons to higher molecular orbitals. The more easily excited the electrons, the longer the wavelength of light it can absorb. Following quantum mechanical selection rules, the molecule will be in a singlet excited state. This technique is complementary to fluorescence spectroscopy. Fluorescence is a relaxation process by which a molecule in the excited state can relax back down to the ground state. In fluorescence, a molecule in the lowest vibration level of a singlet excited state emits a photon to return to the ground singlet state. FTIR is a technique used to obtain an infrared spectrum of absorption or emission of a solid, liquid or gas. An FTIR spectrometer simultaneously collects high-spectral resolution data over a wide spectral range. This confers a significant advantage over a dispersive spectrometer, which measures intensity over a narrow range of wavelengths at a time. The term Fourier-transform infrared spectroscopy originates from the fact that a Fourier transform (a mathematical process) is required to convert the raw data into the actual spectrum.

Nuclear magnetic resonance spectroscopy (NMR) is a spectroscopic technique to observe local magnetic fields around atomic nuclei. The sample is placed in a magnetic field and the NMR signal is produced by excitation of the nuclei sample with radio waves into nuclear magnetic resonance, which is detected with sensitive radio receivers. The intramolecular magnetic field around an atom in a molecule changes the resonance frequency, thus giving access to details of the electronic structure of a molecule and its individual functional groups. 

The aberration-free high-angle annular dark field (HAADF) [29] and scanning transmission electron microscope (TEM) (Z-STEM) [30] are also important tools to analyse the chemical composition of MO/ noble metal nanohybrids because they allow to imaging different elements separately giving a clear understanding of the structural aspects of hybrid structures, particularly for core/multishell structures. Z-STEM is very sensitive to atomic number and, so that, it could be able to achieve atomic resolution making elemental mapping for hybrid structures. These kinds of measurements contribute to understand the physical behaviour of NPs, the conditions for the measurements being really important. Characterizing the final MO/noble metal nanohybrid product is of crucial relevance in order to obtain better insight for both the design and the application of nanohybrids. 

### 2.3. Applications of Magnetic Noble Metal Nanoparticles in Biomedicine

Over the last few years, there has been a steadily growing interest in using nanoparticles in different biomedical applications such as targeted drug delivery, hyperthermia, photoablation therapy, imaging and biosensors. The next section described the application of MO/noble metal hybrid nanoparticles in biomedicine. 

#### 2.3.1. Medical Treatments

**Figure 8:**
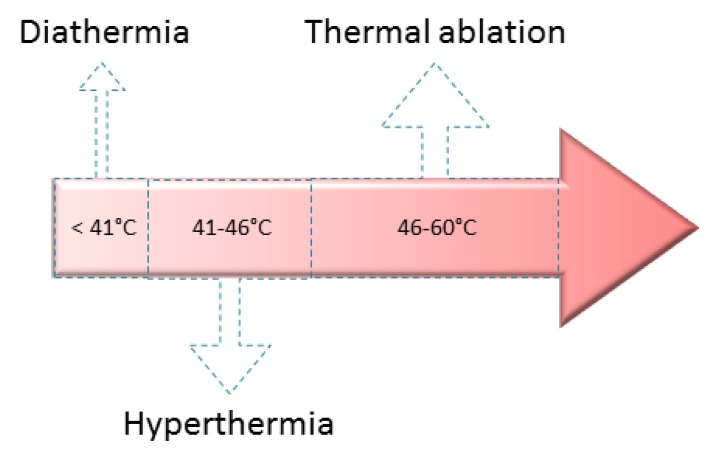
Most commonly employed heating medical treatments.

Among the most commonly employed heating medical treatments thermal ablation, hyperthermia and diathermia could be mentioned [31]. Each of them reaches different temperatures, and by varying the stimulus period of time diverse body results or consequences could be produced [32]. Thermal ablation temperatures usually are between 46–60 °C, whereas hyperthermia and diathermia temperature are near to 41–46 °C and lower than 41 °C, respectively (Figure 8) [33,34,35]. 

Hyperthermia’s main effect on cells is associated with protein denaturalization, leading to the death of the cells involved [36]. It is the most frequently used heating medical treatment and its temperature range destroys cancer cells, but has at the same time a certain insured control of side effects due to healthy cells being less sensitive to this stimulus than tumor ones [14,37,38]. Depending on the organ affected by cancer and the size of the possible treatment area, different kinds of hyperthermia are usually applied: local hyperthermia, regional and whole-body [17,39].

MNPs could be used for hyperthermia treatments if they comply with the required issues, such as selective targeting to cancer cells, their low or null toxicity and precise control of their heating, among many others [40,41]. When the MNPs are in the target area, it is heated by the nanomaterials stimulation through an alternating magnetic field that produces Brownian and Néel relaxation processes [39]. The most widely MNPs employed for hyperthermia are the biocompatible Fe_3_O_4_ and Fe_2_O_3_ iron oxides, and the recommended diameter sizes are in the range 10–40 nm [34,42,43].

Mohammad and collaborators conducted a study in which they compared the amount of heat released by Au-coated superparamagnetic iron oxide NPs (6.3 nm size) regarding neat superparamagnetic iron oxide NPs (5.4 nm size) when low frequency oscillating magnetic fields are applied (44–430 Hz) [44]. The authors found that a 4–5-fold increase in the amount of heat released is achieved in the Au-coated materials. Additionally, under no effects of oscillating magnetic field, both kinds of superparamagnetic iron oxide NPs are not particularly cytotoxic to mammalian cells (MCF-7 breast carcinoma cells and H9c2 cardiomyoblasts) in culture. 

In order to improve the effectiveness of the hyperthermia treatments many efforts are constantly being conducted to optimize the magnetic NPs’ heating efficiency. Instead of this, another strategy could also be considered: the employment of simultaneous magnetic hyperthermia and photothermia [17]. In phototermia, when illuminating certain NPs (such as those containing Au and Ag ones) with light of an appropriate wavelength, a coherent excitation of surface electrons is induced, followed by a rapid relaxation that generates local heat [17]. Since Au and Ag are not approved materials by the Food and Drug Administration (FDA), the employment of these metals as cores being coated by biocompatible materials such as iron oxides could substantially mitigate this problem. In this sense, Das and collaborators have address this challenge and developed nanocomposites constituted by clusters of Fe_3_O_4_ arranged like the petals of a flower around an Ag core, being prepared through a one-step solvothermal process [45]. The researchers obtained promising results: by combining the magnetic hyperthermia properties of Fe_3_O_4_ with the photo-thermal response of Ag the heating efficiency of Ag(core)/Fe_3_O_4_(shell) nanoflowers has been greatly enhanced.

**Figure 9:**
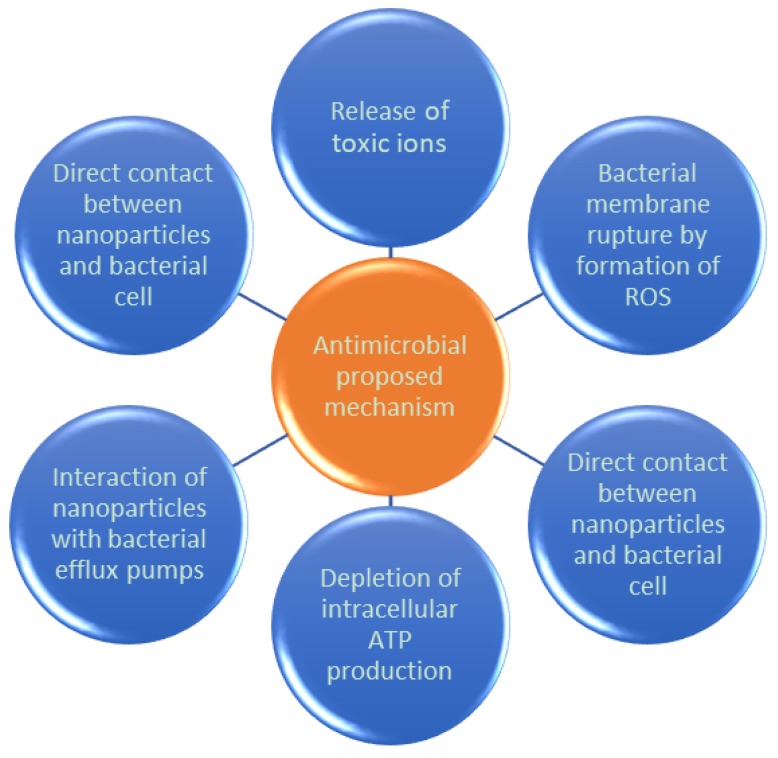
Proposed action mechanisms for the antimicrobial activity of nanoparticles (NPs).

On the other hand, the antimicrobial activity of NPs could be considered as an emerging application that it is still under their first developing stages. The mechanism involved is under discussion, thus six main proposals are being considered: (1) release of toxic ions; (2) bacterial membrane rupture by formation of reactive oxygen species (ROS); (3) direct contact between nanoparticles and bacterial cell (degrading the cell wall and peptidoglycan layer); (4) ROS degrading DNA, RNA, and proteins; (5) interaction of nanoparticles with bacterial efflux pumps; and (6) depletion of intracellular adenosine triphosphate (ATP) production (Figure 9) [46].

For antibacterial purposes, the mostly employed NPs include Ag and Au ones [46,47]. In order to avoid environmental pollution after metal medical treatments, the easy recovery of the nanomaterials is needed and it can be achieved through the incorporation of a magnetic component. Colloidal suspensions of core-shell Fe_3_O_4_-Ag NPs that can be easily recovered by using an external magnet constitute promising nanosystems. In this sense, Chudasama and coworkers presented a one-pot preparation method to obtain narrowly dispersed core shell nanostructures of Fe_3_O_4_-Ag through thermal decomposition of Fe(acac)_3_ [48]. The silver coating of the magnetite nanoparticles thus obtained is formed by the corresponding direct reduction of AgNO_3_. The authors tested the colloid activity against Gram positive and Gram negative bacterial strains. The most pronounced effects were found towards *E. coli* (Gram negative).

Since many medical treatments have reduced efficiency due to the therapeutic agents have no capability to identify diseased cells from healthy ones, many efforts in nanomaterials research are being conducted in this direction. Specifically, bioconjugation refers to a complex formed by the linking of a biomolecule to other biomolecules or small molecules. Some of the most common biological entities involved are proteins, glycans, peptides, enzymes, antibodies, nucleic acids, lipids, carbohydrates and oligonucleotides [49,50]. Bioconjugation research and improvements are important within the medical, diagnostics, microelectronics, life and material sciences fields [51]. In this sense, Xu, Wang and Su reported a short communication about the preparation of Dumbbel-like Au- Fe_3_O_4_ NPs and their employment as Pt delivery devices into Her2-positive breast cancer cells [52]. In accordance to the obtained results, promising results were obtained in terms of NPs capability to act as a multifunctional platform for target-specific platin delivery through strong antibody-antigen interactions.

#### 2.3.2. Medical Diagnosis

Among the available medical diagnosis tools the medical imaging is one of the most widely employed procedures since it allows observing internal parts of the body. In order to obtain the graphic illustrations of the commonly hidden parts of a body, such as organs, specific equipment is required. The techniques commonly used for medical imaging include thermography, fluorescence, medical ultrasonography, X-ray radiography, tactile imaging, computerized tomography (CT), magnetic resonance imaging (MRI), single-photon emission computed tomography (SPECT), photoacoustic tomography (PAT) and optical imaging [53,54]. 

There is a microscopy that can image biochemical processes at subcellular resolution in vivo: the two-photon excitation microscopy (TPM) [55]. This technique allows discovering the details of biological processes and the possible impact of disease and therapies. In the involved optical process fluorophores are excited by simultaneous absorption of two infrared photons, followed by relaxation where a single photon of higher energy in the visible spectrum is emitted [56]. Some noble metals, such as Au and Ag, have been used as optical markers in scattering-based imaging modalities. Jiang and collaborators reported the preparation of bifunctional materials capable to be successfully imaged when they are labelled with live cells by TPM. Additionally, they can be manipulated by employing a permanent magnet, and the researchers consider that the heterodimer nanocomposite could also be useful as contrast agent for MRI applications. The hydrophobic heterodimer nanoparticles were simple prepared by growing Ag nanocrystals with tunable sizes on Fe_3_O_4_ superparamagnetic NPs. 

Magnetic resonance imaging (MRI) works with the employment of both magnetic field and radio waves to produce the desired images [53]. The main advantages of MRI include its ability to distinguish soft tissues and also its very high spatial resolution [57]. When the magnetic field force increases, the resolution and/or the MRI speed also does so [58]. For example, at 3T it is possible to resolve details of the brain as small as 1 mm whereas at 7T it is possible to discern the functional units inside the human cortex (0.5 mm resolution). Furthermore, the world’s most powerful MRI scanner (21.1 T) allowed the determination of the sodium concentration in rat brain tumours, a factor indicating how resistant to chemotherapy it would be [59]. 

To enhance the sensitivity of the MRI technique the selection and use of an efficient contrast agent plays a key role [53]. The contrast material may be administered intravenously or orally (depending on the subject of interest) and it should comply with certain biocompatibility, sensitivity and biodistribution profile characteristics [60], which can be conveniently modelled thanks to nanotechnology and its most recent advances. A very complete summary of the classification and properties of contrast agents for MRI was made by Geraldes and Laurent [57]. Among the inorganic nanomaterials commonly used as contrast agents for medical imaging, iron oxide nanoparticles composed by magnetite (Fe_3_O_4_) or maghemite (γ-Fe_2_O_3_) constitute a very robust and versatile option [53,61,62]. Geraldes and Laurent have summarized iron oxide-based materials currently employed in clinical uses and also describing their commercial names and main characteristics [57]. 

Bimodal imaging diagnosis efficiently combines two techniques leading to improved diagnostic information through synergistic exploitation of their individual advantages. Some of the developed combinations include MRI/ positron emission tomography (PET), CT/MRI, CT/PAT, MRI/SPECT and SPECT/CT [54,63,64,65]. Then, to take advantage and maximize the results offered by this combined techniques, the use of multimodal contrast agents is recommended. In this sense, Wang and coworkers developed folic acid functionalized multimodal contrast agents based on Au nanocages and ultrasmall Fe_3_O_4_ NPs [66]. Authors incorporated the folic acid to the nanoparticles in order to improve their targeting selectivity since the materials based on Au nanocages and ultrasmall Fe_3_O_4_ NPs without the folic acid had no selectivity toward tissues and they were unable to discriminate between malignant and non-malignant tumors. The researchers were focused on combined scanners, specifically those including MRI and CT, since they offer more precise diagnostic tools. For example, in a tumor contour and localization, the MRI gives a comprehensive high spatial resolution of soft tissue information, while the CT offers the real-time and three-dimensional high spatial resolution of hard tissue information. As a result of their research, Wang and coworkers found that the materials designed presented small average size, excellent biocompatibility and low aggregation. Furthermore, they conducted in vitro and in vivo studies that showed long-term circulation time of the nanoparticles, which have renal clearance properties and the capability to be accumulated in tumor tissues. Thus, the presented materials are suitable to be considered as MRI/CT imaging multimodal contrast agents [66].

Other magnetite-based heterostructured NPs were developed and tested as dual model contrast agent for CT/MRI by Zhu and coworkers [63]. Au-Fe_3_O_4_ nanoparticles were obtained by decomposing iron pentacarbonyl onto the surface of Au NPs with further air oxidation of the material. Then, a modification with tetramethylammonium hydroxide was done to produce water-soluble Au-Fe_3_O_4_ NPs. The in vivo probes showed that the as prepared nanomaterials could effectively serve as dual contrast agents. 

Zhao and collaborators also presented Au-Fe_3_O_4_ NPs as dual model contrast agent for CT/MRI [67]. In this case, the synthesis started by the coprecipitation of the magnetite, its further modification with mercaptosuccinic acid (DMSA) and the in situ reduction of HAuCl_4_ to Au nanoclusters of around 1 nm. In vitro and in vivo tests demonstrated that they are potentially useful to distinguish the grade of liver disease.

Li and coworkers prepared Au-Fe_3_O_4_ based NPs by an hydrothermal process as dual model contrast agent for CT/MRI [68]. In the first step, stabilized Au NPs were prepared via the reduction of HAuCl_4_ in the presence of polyethyleneimine (PEI) that was first partially modified with poly (ethylene glycol) monomethyl ether (mPEG) to obtain the mPEG-PEI.NH_2_ conjugate. These NPs were then mixed with Fe^+2^ for the hydrothermal synthesis of the Fe_3_O_4_@Au-mPEG-PEI.NH_2_. Finally, the surface positive charge was reduced by acetylating the remaining PEI surface amines. The researchers verified the dual mode MRI/CT imaging capability of the Fe_3_O_4_@Au NPs after their intravenous injection.

Another dual model contrast agent of CT/MRI based on Au and Fe_3_O_4_ NPs was presented by Cai et al. [69]. The NPs were prepared by these authors first by a coprecipitation method from the Fe^+2^ and Fe^+3^ iron salts to obtain Fe_3_O_4_ NPs. These NPs were then assembled with poly (g-glutamic acid) (PGA) and poly (L-lysine) (PLL) to form PGA/PLL/PGA multilayers. After this, the materials were assembled with dendrimer-entrapped Au NPs formed using amine-terminated generation 5 poly (amidoamine) dendrimers as templates. After crosslinking the multilayered shell of PGA/PLL/PGA/Au dendrimer NPs via 1-ethyl-3-(3-dimethylaminopropyl)carbodiimide hydro- chloride (EDC) chemistry, the remaining amine groups were acetylated, thus neutralizing the surface charge of the particles. Both the in vitro and the in vivo probes demonstrated the NPs potential to be applied for dual mode MRI/CT imaging.

Zhu and collaborators presented bifunctional Fe_3_O_4_-Ag_125_I heterodimers as potential bifunctional contrast agents for MRI/SPECT imaging [54]. The _125_I was included (through rapid reaction with the Ag component of the NPs) due to it being a clinically used radioisotope as a SPECT reporter. Previously, the Fe_3_O_4_-Ag heterodimers were prepared by thermal decomposition of an iron-oleate complex, and then the Ag component was grown onto the magnetite NPs by simple adding the silver acetate. Then, the NPs obtained were PEGylated previously to the reaction with the _125_I at room temperature. The researchers found that the radionuclide NPs showed low cell toxicity and high radiolabelling efficiency. Strong uptake of this nanomaterial by mice liver and spleen was shown by the in vivo SPECT images probes.

An affibody based trimodality nanoprobe (PET; optical imaging; and MRI) for imaging of epidermal growth factor receptor positive tumors was successfully developed by Yang et al. [70]. The dumbbell heteronanostructures were mainly composed by Au and Fe_3_O_4_. Then, authors chose the iron oxide side to be bioconjugated with the selected affibody, and the Au-nanoparticle side was chosen to be modified with a radiometal chelator (NOTA) and low chemical quantity of ^64^Cu. In vitro and in vivo results were promising. 

Zhou and coworkers prepared Fe_3_O_4_@Au NPs potentially useful as multimodal contrast agents for MRI, microwave-induced thermoacoustic and photoacoustic imaging [71]. Firstly, magnetite NPs were prepared through the classical coprecipitation method. The prepared Fe_3_O_4_ NPs were treated with sodium citrate solution, and HAuCl_4_ was then added. The core-shell Fe_3_O_4_@Au NPs, when conjugated with a cancer cell targeted molecular and fluorescent dye, were internalized by the corresponding cancer cells selectively and sensitively, and fluorescence imaging could be done at the same time. Good results were obtained in the multimodal imaging probes. 

#### 2.3.3. Theranostic Agents

A nanoparticle system could have more than one simultaneous application. In the case of NPs for biomedical purposes, the nanomaterial could be conveniently designed to serve for both therapy and imaging applications. These kind of materials are commonly called theranostic agents [72]. For example, iron oxide NPs could be useful as contrast agents for MRI as well as therapeutic carriers, allowing having real-time information of a local treatment whereas it is provided [72]. 

Singh and collaborators prepared Ag@Fe_3_O_4_ core-shell NPs potentially useful as both multimodal imaging and hyperthermia agents [73]. Results showed that the Ag coating intensifies the emission peak corresponding to blue emission, while the Fe_3_O_4_ allows a temperature increase to 40–43 °C in less than 10 minutes of treatment. For the preparation of the magnetic cores coated by Ag a single phase microemulsion method was employed.

Ivashchenko et al. prepared self-organizing silver and iron oxide-based nanocomposites in the presence of Ginger rhizome extract (stabilizer) in their looking for developing a possible theranostic agent [74]. The authors found that the materials prepared through a one-step synthetic methodology presented an almost unique combination of fluorescence, bactericidal and fungicidal properties whereas they also have potential to be an MRI contrast agent. It was found that the ginger rhizome extract not only provided additional fluorescent properties but also it induces hydrocolloids structuring. The nanomaterials preparation was achieved by a variation of the typical coprecipitation method: the iron salts were firstly mixture with the ginger extract. Then, a AgNO_3_ solution also containing the ginger extract was added dropwise. Finally, NaOH was added dropwise to the system, and then the chemicals mixture was heated under reflux for 1.5 h.

Liu and coworkers reported the preparation of FePt@Fe_2_O_3_ core-shell NPs functionalized with polyethylene glicol (PEG) [75]. These NPs could be loaded with doxorubicin (DOX), a chemotherapy drug, in order to achieve targeted intracellular drug delivery and selective cancer cell killing. According to the in vitro and the in vivo results obtained, the presented NPs are promising theranostic agents for cancer cell treatments and MRI diagnosis. Firstly, core FePt NPs were prepared by thermal decomposition from Pt(acac)_2_ and Fe(CO)_5_. Then, a Fe layer was grown by injecting more Fe(CO)_5_, which was then oxidized to form Fe_2_O_3_. The FePt@Fe_2_O_3_ core-shell NPs obtained were further PEGylated and loaded with DOX. 

A variety of Fe_3_O_4_-Au NPs were also developed for theranostic purposes [76]. Han et al. developed a potential surface-enhanced Raman scattering (SERS)-assisted theranostic based on Fe_3_O_4_-Au cluster-shell nanocomposite. This material was tested for free prostate specific antigen (free-PSA) detection, magnetic hyperthermia and MRI, obtaining promising results [77].

Li and collaborators developed Fe_3_O_4_@Au nanorose platform materials capable of offering five different simultaneous applications: dual molecular imaging (MRI/optical imaging), targeting with aptamers and dual therapy (photothermal/chemotherapy) [78]. Briefly, the researchers first prepared Fe_3_O_4_ NPs (by the co-precipitation method from the corresponding iron salts), in which HAuCl_4_ was reduced onto their surfaces to further form the desired nanoroses. Some Fe_3_O_4_-Au core-shell nanocomposites with star shape were also developed by Li et al. for multimodal imaging and photothermal therapy of tumors [79].

Other variety of Au-Fe_3_O_4_ core-shell NPs were designed by Hoskins and coworkers to be used as nano-heaters and also as MRI contrast agents [80]. The iron oxide core is coated with PEI, and the material is then gold-coated via the seeding method. Finally, a thiol (−SH) capped PEG is used to conveniently functionalize the gold surface. The corresponding in vitro studies were conducted in agar phantom gels systems. As a result of irradiation with 532 nm being emitted by a continuous wave laser, at the highest NPs concentration (50 μg·mL^−1^) a ΔT_max_ of 31 °C was observed after the longest tested exposure duration (90 s).

By using two benign moieties (near infrared (NIR) light and nanoshells), Hirsch et al. (2003) [81], have successfully achieved localized and irreversible photothermal ablation of tumor tissue both in vitro and in vivo. In vitro, cells irradiated with very high dosages of NIR laser without nanoshells maintained viability. Likewise, cells incubated with nanoshells in the absence of laser maintained viability as well, suggesting that neither therapy by itself is cytotoxic. Combining these two therapies, however, produced localized cell death confined to the laser nanoshell treatment area. Similar results were seen in vivo. With the aid of magnetic resonance temperature imaging (MRTI), real-time thermal monitoring of tumors treated with the intense NIR-absorbing, non-bleaching nanoshells ensured that successful irreversible thermal destruction was achieved and confined to the tumor volume. Furthermore, histological examination revealed that MRTI estimation of tissue damage was in good agreement with experimental findings, demonstrating its potential utility in determining tissue damage during therapy, making it possible to tailor therapy regimens to ensure the complete thermal destruction of tumors in future studies.

## 3. Conclusions and Future Directions

In this review, it was shown that the synthesis, characterization and applications in biomedicine of MO/noble metal hybrid nanoparticles are really promising fields. The hybridization of two or more metals at nanometric scale could lead to obtain new materials with unexpected, improved and synergic properties (not present on each counterpart). Regarding the obtaining of such kinds of materials, each preparation method displays advantages and drawbacks. Nevertheless, it is important to note that by controlling the variables of synthesis (i.e., mainly temperatures, seeding agents, times, chemical composition, surfactants, reducing agents, preparative methods), several different morphologies of hybrid NPs, such as core/shell-like structures, rattle-type, bricklike, flowers-like, and dumbbell-like NPs, can be produced. The actual tendency in the synthesis of MO/noble metal hybrid NPs is in the direction of a simple design, cost-effective and eco-friendly methods for the production of materials with multifunctional properties. 

Several characterization techniques are useful to analyze the morphology, structure and functional properties of MO/noble metal hybrid nanoparticles. In this area it is important to point out that the use of advanced nanoscale characterization techniques together with computable molecular modeling could provide really useful information for the design of those complex hybrid systems.

Finally, it should be said that many noble metal/iron-based oxide hybrid NPs exhibit potential and promising benefits for both diagnostic and therapeutic medical purposes, as was demonstrated in the review. However, the clinical use of these novel nanometric hybrid materials is still under research since the long-term effects in human bodies are still unknown. The beneficial and harmful effects of this kind of NPs should be deeply studied and compared with other traditional systems.
